# The Complex and Critical Role of Glycine 12 (G12) in Beta-Connexins of Human Skin

**DOI:** 10.3390/ijms22052615

**Published:** 2021-03-05

**Authors:** Rasheed A. Bailey, Derek L. Beahm, I. Martha Skerrett

**Affiliations:** Biology Department, SUNY Buffalo State, 1300 Elmwood Ave, Buffalo, NY 14222, USA; baileyra01@mail.buffalostate.edu (R.A.B.); beahmdl@buffalostate.edu (D.L.B.)

**Keywords:** connexin, skin disease, glycine, gap junction structure

## Abstract

Glycine is an amino acid with unique properties because its side chain is composed of a single hydrogen atom. It confers conformational flexibility to proteins and conserved glycines are often indicative of protein domains involving tight turns or bends. All six beta-type connexins expressed in human epidermis (Cx26, Cx30, Cx30.3, Cx31, Cx31.1 and Cx32) contain a glycine at position 12 (G12). G12 is located about halfway through the cytoplasmic amino terminus and substitutions alter connexin function in a variety of ways, in some cases altering protein interactions and leading to cell death. There is also evidence that alteration of G12 changes the structure of the amino terminus in connexin- and amino acid- specific ways. This review integrates structural, functional and physiological information about the role of G12 in connexins, focusing on beta-connexins expressed in human epidermis. The importance of G12 substitutions in these beta-connexins is revealed in two hereditary skin disorders, keratitis ichthyosis and erythrokeratodermia variabilis, both of which result from missense mutations affecting G12.

## 1. Genes, Proteins and Gap Junction Channels

Connexin proteins are expressed almost ubiquitously in vertebrate tissue where they form gap junction channels and sometimes function as transmembrane channels. The human genome encodes 21 different connexins while the mouse genome encodes 20 connexins, the majority of which have human orthologs [1]. Connexin proteins are expressed in specific and overlapping patterns for instance Cx40 is co-expressed with a group of other connexins (e.g., Cx37) in endothelial cells and with a different group of connexins (e.g., Cx43 and Cx45) in cardiomyocytes [1] The genes that encode connexins are divided into five groups (A, B, C, D and E) based on sequence. Seven of the 21 human connexins are categorized as beta connexins including Cx25, Cx26, Cx30, Cx30.3, Cx31, Cx31.1 and Cx32 [1,2] and of these, all except Cx25 are expressed in the skin [3]. Within the epidermis distinct subpopulations of cells express different combinations of connexins and there is much to be learned about the diversity and function of channels that result from connexin interactions.

**Figure 1:**
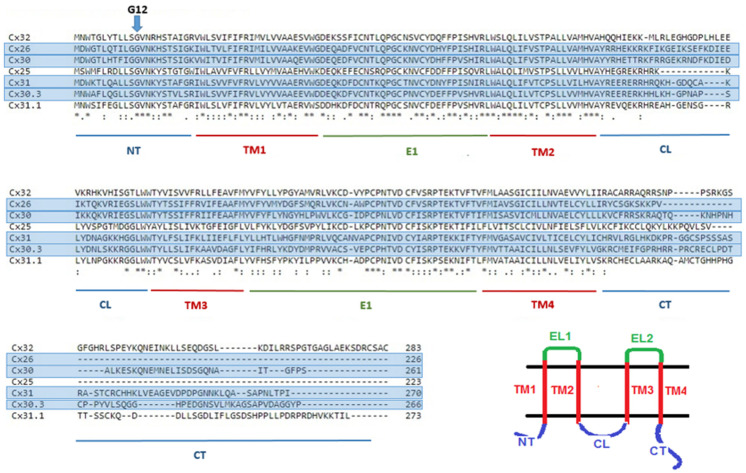
Amino acid alignment of beta-connexins. All include a glycine at position 12 which is located about halfway through the amino terminus and is one of only three conserved glycines. Cx26, Cx30, Cx30.3 and Cx31 (blue highlight) are coexpressed in the stratum granulosum and are associated with hereditary skin disorders. Sequences were aligned using Clustal Omega [4]. Inset: Membrane topology of a connexin protein four transmembrane domains in red (TM1-TM4), two extracellular loops in green (EL1 and EL2) and cytoplasmic domains in blue (NT = amino terminus; CL = cytoplasmic loop and CT = cytoplasmic tail).

Gene names for gap junction proteins include the prefix GJ (Gap Junction), the group (A, B, C, D or E) and a number representing the order in which genes were identified [2]. For example, *GJB1* which encodes Cx32 was the first gene encoding a beta-connexin to be identified. Connexin protein names reflect their predicted molecular weight, for example Cx32 has a predicted molecular weight of 32 kilodaltons. Connexins have four membrane-spanning domains with cytoplasmic amino and carboxyl termini. As shown in the amino acid alignment of human beta connexins (Figure 1) there is a high degree of conservation of sequence within the amino terminus (NT), membrane-spanning domains (TM1-TM4) and extracellular domains (E1, E2). The cytoplasmic loop and cytoplasmic tail (CL, CT) have distinct amino acid sequences.

Each gap junction channel is composed of twelve connexin proteins, six from each cell. In most cases connexins oligomerize in the ER and follow a typical secretory route to the plasma membrane as a closed hexameric channel [5]. Upon insertion in the plasma membrane the channels usually remain closed until they dock with channels in an adjacent cell. Mutations can alter the biosynthetic pathway in a number of ways by introducing defects in trafficking, assembly or gating [6].

## 2. Connexins and the Human Epidermis

**Figure 2:**
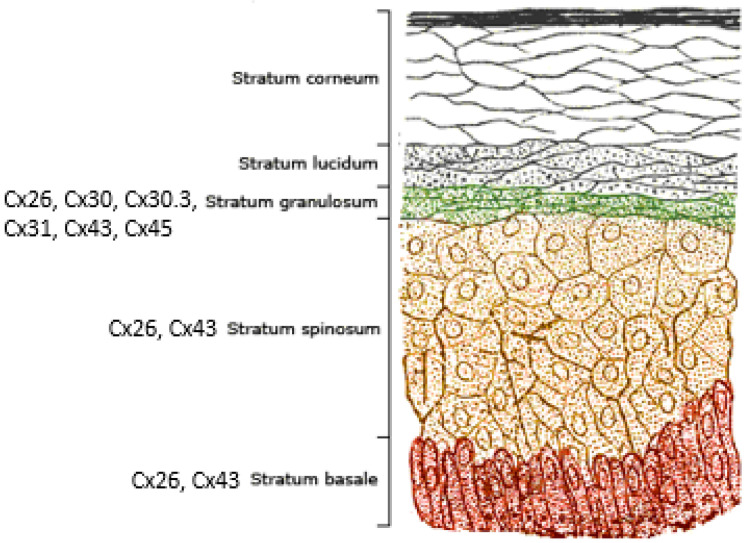
Illustration of human skin showing the progression and differentiation of cells from the stratum basale to the stratum corneum. Beta-type connexins include Cx26, Cx30, Cx30.3, and Cx31 which are co-expressed in the stratum granulosom. In addition, Cx26 is expressed throughout the layers of the epidermis along with Cx43 an alpha-type connexin. Image from Wikipedia.or modified to include connexin expression results from [3].

The human epidermis is the outermost layer of skin that forms a barrier protecting the body from pathogens and prevents dehydration [7]. It is composed primarily of keratinocytes [8] and is organized in layers with the deepest layer, the stratum basale, producing cells that migrate toward the surface, the stratum corneum. A cross sectional representation of human epidermis is shown in Figure 2. Cells in the basal layer divide creating new cells that are in a continuous state of stratification and differentiation until they reach the stratum corneum and are shed. As keratinocytes transition through the layers of the epidermis they express a wide range of junctional and extracellular proteins that contribute to barrier function and tissue strength [9]. Gap junctions are important for both cell adhesion and cell communication in the epidermis, and at least ten connexins are expressed in human epidermis [3,10,11]. Some are broadly expressed while expression of others is limited to specific cell types. For example, Cx26 and Cx43 are expressed throughout the stratum, in the layers basale, spinusom, and granulosum, while Cx30, Cx30.3, Cx31 and Cx45 are expressed only in the stratum granulosum [3].

Thus far, five connexins have been linked to hereditary disorders affecting the skin including Cx26, Cx30, Cx30.3, Cx31 and Cx43 [10,12]. Mutations in the gene encoding Cx26 cause keratitis-deafness-ichthyosis (KID) a potentially fatal condition affecting the eyes, skin and hearing [13]. Mutations in the gene encoding Cx30 cause Clouston syndrome, a disorder that affects hair, teeth, nails and skin [14]. Mutations in the gene encoding Cx30.3 are linked to erythrokeratoderma variabilis (EKV) [15] a disease characterized by transient erythema and hyperkeratosis. Mutations in the gene encoding Cx31 also cause EKV [16]. The only alpha-type connexin so far associated with a hereditary skin disorder is Cx43. Mutations in Cx43 cause occulodental digital dysplasia (OCDD), a developmental disorder affecting skin, nails and teeth with degenerative components that may involve skin [17,18].

## 3. Glycine 12 in Beta-Connexins Is Conserved and Essential

All beta-connexins include a glycine residue at position 12 and mutations that alter G12 are associated with skin disease as well as hereditary forms of deafness and neuropathy. Table 1 summarizes hereditary disorders linked to G12 substitutions in beta-connexins.

**Figure 3:**
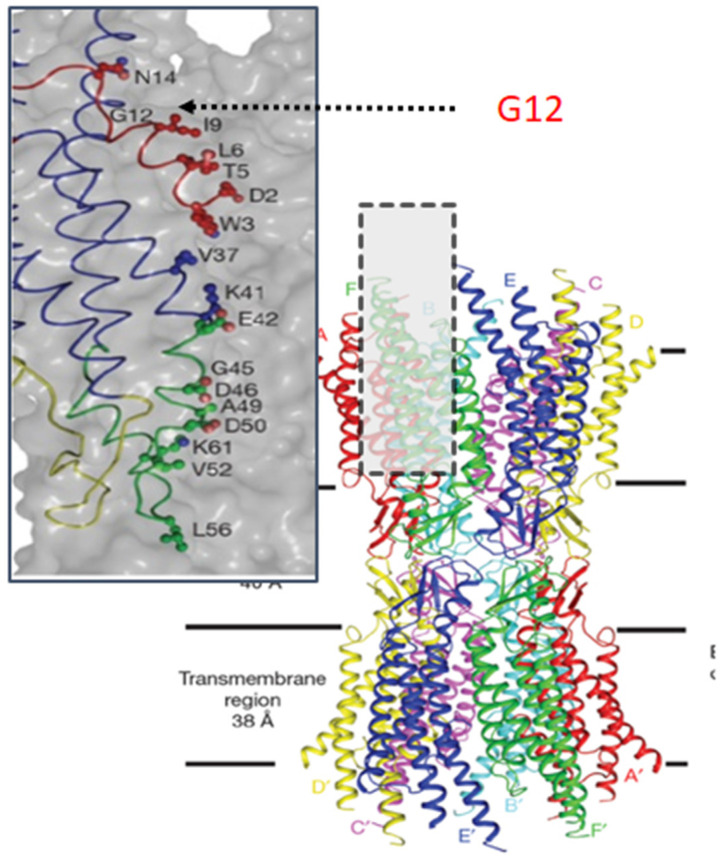
Structure of the Cx26 gap junction channel in ribbon representation. Twelve connexin subunits form a pore that spans two cell membranes and the extracellular space. Inset: Pore structure highlighting location of G12 with the amino terminus (red) which folds into the pore. Modified from [40].

Understanding the consequences of G12 mutations requires knowledge of protein structure, protein interactions and conformational changes that underlie function. The general structure of connexin channels is reasonably well understood and there are several 3D structures available for gap junction channels [38,39,40,41,42]. These structures universally reveal a large aqueous pore spanning two cell membranes and the extracellular space. They also confirm that twelve connexin proteins contribute to each channel, six from each cell and that the intercellular pore is lined by parts of the transmembrane domains, extracellular loops and amino termini. Three dimensional structures for Cx26 [39,40,41] are the only representatives of beta-type connexins. The amino terminus was first revealed in the electron crystallographic study by Oshima et al. [39] (2007) at 10–14 Å resolution which revealed a plug in the inner vestibule of the channel, suggesting that the amino terminus folds into the mouth of the pore. There was some uncertainty surrounding the plug because a point mutation (M34A) induced in the Cx26 protein prior to crystallization results in channels that reside in a closed state [43] and it was therefore unclear whether the plug was specifically related to a closed state of the channel [39]. The 3.5 Å resolution X-ray crystallographic analysis by Maeda et al. [40] revealed the amino terminus folded into the cytoplasmic mouth of the pore and revealed interactions between the amino terminus and those within the first transmembrane domain. Since the mutation M34A was not induced prior to crystallization it is now generally accepted that the amino terminus resides within the mouth of the pore when the channel is open. A side-view of a Cx26 gap junction channel is shown in Figure 3. The N-terminus (NT) is highlighted in red (inset) and includes an open turn between residues 12 and 15. This turn is likely important in positioning the N-terminal within the mouth of the pore.

## 4. Understanding the Role of G12

Despite informative structural data [40] and ample evidence that glycine facilitates turns in proteins [44] identifying the structural and functional malfunctions that underlie disorders resulting from mutations of G12 is complicated. The amino terminus plays important roles in oligomerization, protein trafficking, channel gating and permeability [45,46,47,48,49,50]. However, there is a lack of consistency regarding the importance of specific residues and structural elements, likely related to intricate differences in the structure and function of connexins. Each of the 21 connexins imparts unique structural and functional properties on the channels to which it contributes. Each connexin selectively interacts with other connexins and other cellular proteins while responding to cellular signals that regulate channel behavior. It has become apparent that the disruption of G12 causes changes that are connexin- and circumstance- specific, a logistical complexity that may best be addressed using thermodynamic models [51,52]. Currently, the best insight into N-terminal structure and the role of G12 in beta-connexins comes from NMR studies of N-terminal peptides.

## 5. N-Terminal Peptides

The first NMR analysis of an N-terminal peptide was published in 2000 [53] and involved amino acids 1–15 of Cx26 [53]. The peptide included two structured domains connected by a flexible hinge with residues 12 through 15 forming an open turn. Purnick and colleagues [53] reported that the first ten residues of the Cx26 NT domain adopt a helical conformation while residues 12–15 form an open turn. Purnick and colleagues [53] referred to the region surrounding G12 as a “domain–hinge–domain” motif. Importantly, the NMR structure of the Cx26 NT is similar to the structure of the NT in the 2009 structure of Cx26 published by Maeda and colleagues [40]. Consistent with NMR interpretation regarding flexibility around Maeda et al. [40] reported that the amino terminus was one of the most mobile domains in the structure.

In 2009 Kalmatsky et al. [49] applied the same NMR methodology to the study of peptides corresponding to the amino terminus of Cx32. Mutations with clinical significance (linked to CMTX) were studied with regard to their effect on the unconstrained turn associated with G12. Many of the mutants had previously been assessed for function [46,53] in paired oocytes. Using NMR Kalmatsky et al. [49] showed that mutations that altered the open turn around residue 12 (e.g., G12S, G12Y) were correlated with loss of function while G12P retained flexibility and function.

In 2016 Batir et al. [54] further examined N-terminal peptides focusing on the incorporation of mutations that cause disease. They focused on the G12R mutation in Cx26 and Cx32. Interestingly, the substitution to arginine induced greater flexibility in Cx26 but a more constricted turn in the Cx32. This highlights the importance of connexin- and mutation-specific analysis as mutations at position G12 alter the structure of amino terminal peptides in ways that are specific to both the connexin and the amino acid.

## 6. Functional Analysis of G12 Mutants

The wide range of functional effects resulting from substitutions at G12 can be broadly classified as loss-of-function or gain-of-function. Loss-of-function mutants are unable to perform their cellular duties because they do not function optimally. They are further grouped based on their functional defect (e.g., trafficking-deficient, altered oligomerization, rapid turnover). Gain-of-function mutants take on new and destructive roles in a cell. Two important types of gain-of function mutants are those that exert their effects through protein interactions and those that result in poorly regulated membrane channels.

**Figure 4:**
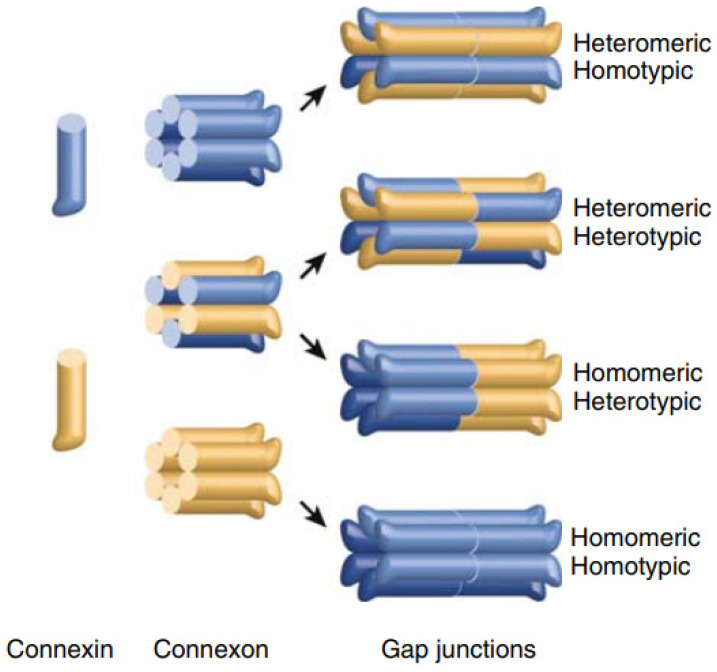
Illustration of connexin interactions resulting in heteromeric and heterotypic channels. When more than one connexin is expressed in a cell the connexins may oligomerize to form a heteromeric connexon. If the connexon functions as a transmembrane channel, as occurs in a small number of cases it is referred to as hemichannel. It is generally assumed that connexins from the same group are capable of forming heteromeric channels although very little information is available about this process. When connexons from different cells interact there is an almost limitless number of possible gap junction channel compositions the simplest being homomeric homotypic (bottom right) and the most complex being heteromeric heterotypic (top right). Most structural and functional analyses have focused on homomeric and homotypic channels. Image modified from [57].

Only a few connexin mutations have been shown to induce gain-of-function by altering interactions with other proteins [23,55]. As at least one mutation involving G12 falls into this category it is important to understand basic connexin interactions. Most cells express more than one connexin and connexins intermingle within and between cells, typically with others from the same group (e.g., beta-connexins interact with other beta-type connexins). Heteromeric channels form when different connexins from the same cell oligomerize and heterotypic channels form when different connexins interact between adjacent cells. Figure 4 summarizes connexin interactions resulting in heteromeric and heterotypic channels. Given the broad overlapping expression patterns of connexins this intermingling is presumed to underlie almost limitless combinations of channels.

The other type of gain-of-function mutant relevant to studies of G12 is that associated with unregulated channel activity. As mutations at G12 may fall into this category it is important to understand the types of channels formed by connexins. Most connexins oligomerize in the ER and traffic to the plasma membrane as hexameric channels. Prior to the formation of gap junction channels, these hemichannels (also known as connexons, Figure 4) aggregate at the plasma membrane and remain closed prior to docking [56]. Pathogenic mutations can disrupt regulation, leading to cellular dysfunction or death.

In summary, G12 mutations cause amino-acid and connexin-specific changes in function. These changes in function underlie disease physiology and are broadly categorized as gain-of-function or loss-of-function. Understanding the functional consequences of G12 mutations in detail may lead to treatment of diseases as therapies become available that target cellular processes such as protein trafficking or channel regulation. Specific information related to skin diseases caused by G12 mutations in beta-connexins is provided below with information, where available regarding the functional consequences of mutations at the cellular level.

## 7. Connexin 26 (GJB2)

Mutations in GJB2 are associated with several skin disorders including keratitis-ichthyosis-deafness (KID) syndrome [13] a severe skin disorder that also presents with sensorineural hearing loss [58]. Both hereditary and sporadic cases of KID syndrome have been reported; the hereditary form is linked to dominant mutations in GJB2. This is in contrast to the hundreds of recessive mutations in GJB2 that cause sensorineural hearing loss without consequences in skin [12,59,60].

In Cx26 two mutations resulting in amino acid substitutions at G12 have been linked to disease. The mutation G12R is associated with keratitis-ichthyosis-deafness (KID) syndrome [25,26,61] while the mutation Cx26G12V is associated with sensorineural deafness [21,62]. These two mutations are often described as syndromic and non-syndromic, respectively. When expressed in cells the mutants behave very differently in ways that are consistent with the severity of their physiological consequence.

Cx26G12R has gain-of-function properties although the full extent of these has taken some time to uncover. Over ten years ago Cx26G12R was expressed in *Xenopus* oocytes where it failed to form gap junctions suggesting a loss-of-function at the level of gap junction intercellular coupling [25]. In the same study it was found to induce currents in single oocytes suggesting an additional gain-of-function mechanism at the hemichannel level. Lee and colleagues [27] observed that oocytes injected with RNA encoding Cx26G12R were susceptible to cell death (to a greater extent than those injected with Cx26 RNA). Consistent with hemichannel activity, cell death was rescued by increasing the concentration of calcium in the external media from 0 mM to 4 mM. These results are indicative of gain-of-function at the hemichannel level although several other mechanisms could explain the observations such as cell toxicity due to trafficking defects and/or induction of the unfolded protein response. This study involved expression in *Xenopus* oocytes, an excellent model system for assessing mutations at the biophysical level, but not ideal for studies of trafficking, other groups sought to assess the properties of Cx26G12R in mammalian cells.

The mutant Cx26G12R was subsequently expressed in HaCaT cells, a model human keratinocyte cell line [63]. Due to their interest in the inflammatory nature of KID syndrome, Donnelly and colleagues focused on ATP release stimulated by peptidoglycan isolated from skin bacteria. Peptidoglycan was applied to HaCaT cells and connexin-deficient HeLa cells. As hemichannels are permeable to metabolites hemichannel activity was assessed by monitoring ATP release. By studying ATP release in cells expressing KID mutants (e.g., Cx26G12R) and non-KID mutants Donnelly and colleagues [63] found that Cx26G12R mediated ATP release at levels above those of wildtype. They also found that ATP release was blocked by carbenoxolone, a non-specific blocker of gap junctions and hemichannels. They found no evidence of ATP release across the membrane of cells expressing non-KID mutants [63]. This study provided strong evidence that the leak currents and cell death associated with expression of Cx26G12R in oocytes results from aberrant hemichannel behavior.

The KID syndrome mutation Cx26G12R has also been correlated with gain-of-function in studies examining oligomerization. Being a beta-type connexin, Cx26 was not expected to interact with the alpha-type connexin Cx43. García et al. [23] showed that when the Cx26G12R mutant was co-expressed with Cx43 it formed heteromeric channels. While surprising this observation was consistent with a previous report that G12 plays key role in oligomerization compatibility of alpha and beta type connexins [64]. The hemichannels formed by the oligomerization of Cx26G12R and Cx43 induce leak currents in the cell membrane and are also unable to form functional gap junction channels [23]. Combined with earlier studies the work by Garcia and colleagues [23] this suggests that gain-of-function related to the mutant Cx26G12R is two-fold because (i) the mutant interacts with Cx43 atypically and (ii) results in hemichannels that mediate ATP release and calcium overload. In addition Cx26G12R displays loss-of-function related to the formation of intercellular channels [23,25].

A more biophysical characterization of Cx26G12R [65] focused on channel gating and potential interactions between the amino terminus and the cytoplasmic loop. Using a range of techniques including MD simulations the authors confirmed that G12 is involved in gating and calcium regulation. This study is of particular interest to those attuned to the intricacy of gap junction channel gating as it points to disruption of the mechanism involved in Vj (transjunctional) gating rather than loop gating as a cause for Cx26G12R gain-of-function [65].

There is less information about the behavior of the deafness mutant Cx26G12V. This mutant is associated with mild hearing loss except when the allele is heterozygous with a truncating mutant such as 35DelG [66]. Snoeckx and colleagues [66] proposed that Cx26G12V likely results in a defect in intracellular trafficking, like other non-truncating deafness mutants, but also emphasized that GJB2 deafness mutants, particularly the amino acid substitution mutants, reveal a wide range of often contradictory outcomes. Garcia and colleagues [23] studied a GFP-tagged version of Cx26G12V in a study related to KID mutations. In their analysis Cx26G12V trafficked to the membrane and formed gap junction plaques with similar size and distribution to plaques observed in cells expressing wtCx26-GFP. The authors also noted that G12V oligomerized with wildtype Cx26, did not form heteromeric channels with Cx43, and was not associated with aberrant hemichannel activity [23].

## 8. Connexin 30 (GJB6)

Cx30 is expressed in epithelial cells of the skin, specifically in the stratum granulosum [3]. It is also expressed in epithelial and mesenchymal cells of the inner ear [67] and similar to Cx26, mutations in Cx30 can cause both skin disease and deafness [68]. The skin disorder associated with mutations in Cx30 is an autosomal dominant condition known as Clouston syndrome which is characterized by abnormalities of the hair follicles, nails, and skin [14]. Mutations involving G12 have not been reported but an adjacent glycine (G11) is implicated in Clouston syndrome [69]. Glycine occurs at position 11 in only two of the beta-type skin connexins (Cx26 and Cx30) while other beta-type connexins of skin include serine at position 11 (e.g., S11, G12).

One study of Cx30G11R reported that this mutant trafficked to the membrane and formed gap junction plaques that facilitated dye transfer between cells. They also reported that the mutation induced a gain-of-function through the formation of hemichannels that mediate ATP release [70]. This is consistent with studies of mutations at the adjacent G12 and their role in connexin-related skin disease.

## 9. Connexin 30.3 (GJB4)

Another beta-type connexin of skin that includes a mutation at position G12 is Cx30.3. Mutations in the gene encoding connexin30.3 are linked to the hereditary skin disease erythrokeratodermia variabilis (EKV) [15] a disease characterized by transient erythema and hyperkeratosis. The mutation Cx30.3G12D was first associated with skin disease in 2003 [15,29]. Thus far, there are no published reports related to the function of Cx30.3G12D.

Future analysis of Cx30.3 mutations, including G12D should involve studies of connexin interactions particularly with Cx31. These two connexins are co-expressed in the stratum granulosum and although EKV spans diverse phenotypes and symptoms, there is much similarity between the outcomes of mutations in Cx30.3 and Cx31. Plantard and colleagues [71] studied interactions between Cx30.3 and Cx31 and found that the two connexins oligomerize to form heteromeric channels and that the oligomerization increases the size of gap junction plaques. As evidence that mutations can influence interactions between Cx30.3 and Cx31, the mutation Cx30.3 F137L resulted in decreased coupling based on a trafficking defect, the functional consequences of which further decrease functional Cx31 gap junctions. This suggests that Cx30.3 mutations may act in a trans-dominant manner, reducing the function of interacting partners such as Cx31.

## 10. Connexin 31 (GJB3)

GJB3 was the first gap junction gene linked to hereditary skin disease and mutations cause erythrokeratodermia variabilis (EKV), a disease characterized by transient erythema and hyperkeratosis [16]. Mutations in GJB3 can also be associated with hereditary deafness with or without neuropathy [72]. Both dominant and recessive mutations in GJB3 are associated with EKV. The two EKV mutations involving amino acid substitutions at position G12 are both dominant [16] (Cx31G12R and Cx31G12D).

A number of studies investigating the outcomes of Cx31G12R and Cx31G12D in cells have been conducted. While all studies have identified alterations in protein behavior, the studies have produced variable results. Part of the variability may stem from the fact that replacement of glycine 12 with different amino acids (arginine versus aspartate) affect channel formation and function differently. However, experiments with the same mutants have produced different results when conducted in different cell lines and using different methods of expression and analysis. This suggests that expression levels and interacting proteins may underlie some of the differences.

Di and colleagues [30] expressed mutants Cx31G12D and Cx31G12R (fluorescently tagged with EGFP) in NEB cells and reported that both mutants accumulated in the cytoplasm/endoplasmic reticulum. Cells expressing the mutants tended to round up and die suggesting that Cx31G12D and Cx31G12R experience trafficking malfunctions that induce UPR and cell death. In contrast other Cx31 mutations associated with deafness/neuropathy did not induce cell death. When Diestel and colleagues [73] expressed Cx31G12R in HeLa cells they observed a similar result as cells expressing the mutant experienced higher death rates. By using an inducible expression system the authors were further able to demonstrate a correlation between cell health and the expression level of Cx31G12R [73]. These results combined with those of Di and colleagues [30] suggest Cx31G12R and G12D mutants induce cell death. However, unlike the study by Di and colleagues [30], Diestel’s group observed that cells expressing Cx31G12R were dye-coupled and reported that dye-coupling occurred to a greater extent in G12R-expressing cells than in cells expressing Cx31 [73]. This contradicts the observation that trafficking is affected by the G12R substitution and suggests that the mutant may induce a gain-of-function at the hemichannel level causing leaky cells and cell death and also a gain of function at the gap junction level.

Rouan and colleagues [32] expressed Cx31G12D in connexin-deficient HeLa cells where the mutant was found to oligomerize and traffic to the membrane but did not form functional gap junction channels in dye-coupling experiments [32]. Cell death was not reported in this study.

In contrast, He and colleagues [33] expressed Cx31G12D and Cx31G12R in HeLa cells and used a fluorescent localization assay to assess gap junction formation. They reported reduced plaque formation in cells expressing both mutants suggesting defects in trafficking or assembly. They also observed cell death in cells expressing Cx31G12R and Cx31G12D. These observations were particularly interesting because they contrasted the effect of a recessive Cx31 mutant (L34P) as well as a group of Cx31 mutants associated with hearing impairment but not skin disease (66DelD, 141delI, R180X and E183K). All of these failed to form gap junction plaques and did not induce cell death.

Tattersal and colleagues [74] similarly reported that expression of Cx31G12D induced cell death whereas Cx3166DelD did not. A more thorough analysis of ER stress was carried out and it was determined that cell death was associated with induction of the unfolded protein response (UPR).

**Figure 5:**
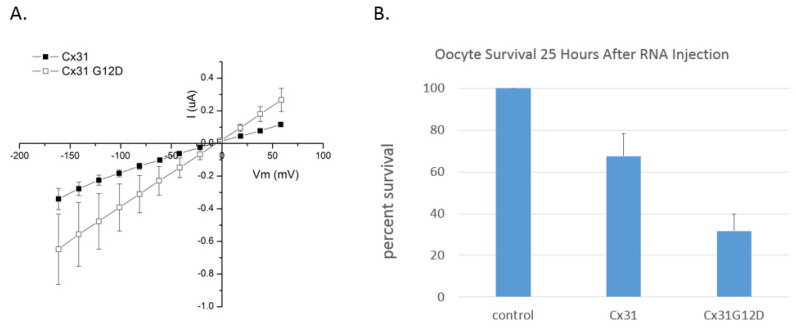
(**A**) Cx31G12D induces membrane currents in *Xenopus* oocytes. Current versus voltage plots for Cx31G12D and wildtype Cx31 demonstrate that the mutant G12D increases membrane permeability. Currents were induced by 20 mV voltage pulses from a holding potential of −20 mV, *n* = 3. Image from [76] Bailey, 2020. (**B**) Cx31G12D induces cell death in single oocytes. Oocytes were assessed for survival 25 h after injection of RNA. Those extruding cytoplasm were considered dead. Since there was no apparent effect of divalent cations on cell survival results were pooled revealing that 32% of oocytes expressing Cx31G12D survived compared to 68% of control Cx31-injected oocytes and 100% of oocytes injected with antisense oligonucleotide against XeCx38.

Unlike the Cx26 G12 mutants there is no published information regarding the expression of Cx31G12D or Cx31G12R in *Xenopus* oocytes. This type of analysis would provide a more direct assay for leaky membranes and gain-of-function hemichannels. This type of analysis has been conducted for another Cx31 skin disease mutant (e.g., R42P) and results support disruption of hemichannel regulation as a cause of cell death [75]. As part of a project involving expression and characterization of Cx31 mutants in our lab, Bailey [76] studied behavior of oocytes expressing wildtype Cx31 and Cx31G12D. He noticed that the mutant induced higher membrane currents than Cx31 (Figure 5A). Cx31G12D also had a negative impact on oocyte health, and survival was not enhanced by increasing the concentration of calcium or cobalt in the external solution. For instance, 25 h after injection almost 70% of oocytes expressing Cx31 survived regardless of external calcium concentration while just over 30% of Cx31G12D expressing cells survived (Figure 5B). This further supports a gain-of-function role for Cx31G12D.

## 11. Connexin 31.1 (GJB5)

Cx31.1 is expressed in human skin and the inner ear [77] (Xia et al., 1998). Mutations are associated with deafness but so far not skin disease. Additionally, no mutations have so far been identified at position G12.

## 12. Connexin 32 (GJB1)

Thus, far there have been no skin diseases associated with mutations in Cx32, possibly because it is expressed at low levels and only in specific types of skin (e.g., the palm, [3]). Cx32 mutations cause a neurodegenerative disease known as Charcot-Mare Tooth Disease type X (CMTX) [78]. Hundreds of mutations have been identified [34] and one of these involves G12 (G12S) [36,37]. Like many of the CMTX mutations in Cx32, G12S exhibits a defect in trafficking leading to intracellular accumulation of the connexin [35,79]

## 13. Conclusions

Glycine12 (G12) of beta-connexins is positioned midway through the cytoplasmic amino terminus (NT) where it contributes to conformational flexibility allowing the NT to fold into the mouth of the pore [40,53]. It also plays a role in connexin oligomerization [5,64]. In three of the beta-connexins expressed in the epidermis (Cx26, Cx30.3 and Cx31) substitutions at G12 are associated with skin disease. Cx26G12R is associated with keratitis-ichthyosis-deafness (KID) syndrome [25,26,61] while Cx30.3G12D, Cx31G12R and Cx31G12D are associated with erythrokeratodermia variabilis [15,16]. The importance of this residue is further evident in the identification of G12 mutations associated with sensorineural deafness (Cx26G12V), Charcot-Marie-Tooth disease (Cx32G12S) and oculodentodigital dysplasia (Cx43G12R) [80]. While it is common to find sensitive residues linked to two connexin disorders such as M34T which is associated with CMTX in Cx32 and deafness in Cx26 [80], it is rare to find mutations of the same residue linked to five disorders. Additionally, of interest is the wide range of outcomes after amino acid substitutions, both genetically and in cellular expression studies [10,12,80]

It is not surprising that replacement of glycine disrupts function, and certainly mutation of other glycine residues in connexins can cause disease. For example, Cx26G11E and Cx26G45E are both associated with skin disease [80,81]. The surprising aspect of G12 mutations is the complex and sometimes conflicting behaviors they exhibit when expressed in cells. The mutation G12R in Cx26 not only disrupts gap junction intercellular communication [23,25] but induces new connexin interactions that lead to leaky hemichannels, ATP release and calcium overload [23,25,63,64]. Cx31G12D and Cx31G12R have been linked to a variety of outcomes the most common being cell death [30,33,73,74,76] attributed to trafficking defects and induction of UPR [30,74], In some studies membrane localization and/or gap junction intercellular communication were reported in cells expressing Cx31G12D or Cx31G12R [32,73]. The conflicting results may be related to background expression of Cx43 which was shown to oligomerize with Cx26G12R [23,24]. Interestingly Cx43 contains an arginine at position 12 (R12) and this residue is one of only a few amino acids determined to be critical for oligomerization [64]. Cx26G12R, a dominant KID mutation, exerts dominant effects at the cellular level via interaction with Cx43. All of the G12 mutations associated with skin disease appear to be dominant [25,26,61], supporting the possibility that they impart trans-dominant properties. In keeping with this observation, less severe substitutions at G12 of beta-connexins are associated with recessive disorders (e.g., Cx26G12V NSHL; Cx32G12S; CMTX). In light of these findings connexin interactions should be assessed for other G12 mutants.

## Figures and Tables

**Table 1:** Hereditary Disorders Associated with G12 Mutations in Beta-Connexins.

Connexin (Gene)	Disorder	Background	G12 Mutation
Cx26 *(GJB2)*	Deafness Non-syndromic	Over 300 recessive mutations, mostly point mutations. 35DelG with truncation at amino acid 13 is most prevalent [19,20]	G12V [21,22,23]
	Skin Disease with Deafness Keratitis ichthyosis and deafness (KID) syndrome Rare skin disorders with or without inflammatory response	Numerous dominant point mutations, sporadic and hereditary. Focused in NT and E1 domains [12,24] Dominant point mutations throughout coding region [12]	G12R [24,25,26,27]
Cx30 *(GJB6)*	Skin Disease Clouston syndrome	A few point mutations resulting in amino acid substitutions in various regions [12]	
Cx30.3 *(GJB4)*	Skin Disease erythrokeratodermia variabilis et progressiva (EKV)	A few point mutations resulting in amino acid substitutions in various regions [15,28]	G12D [15,29]
Cx31 *(GJB3)*	Skin Disease erythrokeratodermia variabilis et progressiva (EKV) Hearing and neurological disorders	At least 20 mutations, most dominant and appear to result in trafficking defects and cell death [30] Several mutations associated with deafness and one with neurological disease [31]	G12D [32] G12R [33] G12S [31]
Cx32 *(GJB1)*	Neurodegenerative X-linked Charcot-Marie-Tooth (CMTX) disease	Hundreds of recessive mutations, mostly point mutations in all regions of protein [34]	G12S [35,36,37]
